# Chitosan treatment reduces softening and chilling injury in cold-stored Hami melon by regulating starch and sucrose metabolism

**DOI:** 10.3389/fpls.2022.1096017

**Published:** 2022-12-14

**Authors:** Qin Zhang, Fengxian Tang, Wenchao Cai, Bo Peng, Ming Ning, Chunhui Shan, Xinquan Yang

**Affiliations:** ^1^ College of Food, Shihezi University, Shihezi, Xinjiang Uygur Autonomous Region, China; ^2^ Engineering Research Center of Xinjiang Characteristic Fruit and Vegetable Storage and Processing, Ministry of Education, Shihezi University, Shihezi, Xinjiang Uygur Autonomous Region, China; ^3^ School of Life Sciences, Guangzhou University, Guangzhou, Guangdong Province, China

**Keywords:** chilling injury, chitosan, cold storage, melon softening, starch and sucrose metabolism

## Abstract

Cold-stored Hami melon is susceptible to chilling injury, resulting in quality deterioration and reduced sales. Pre-storage treatment with chitosan reduces fruit softening and chilling injury in melon; however, the underlying mechanism remains unclear. In this study, Gold Queen Hami melons were treated with 1.5% chitosan solution for 10 min before cold storage at 3°C and then the effect of chitosan was examined on fruit firmness, weight loss, chilling injury, soluble solid content (SSC), pectin, and soluble sugar contents of melon fruit. Also, the enzyme activities and gene expressions related to fruit softening and starch and sucrose metabolism were investigated. Chitosan treatment reduced the fruit softening and chilling injury, maintained the high levels of starch and sucrose contents, and regulated the enzyme activities and gene expressions related to starch and sucrose metabolism. Fruit firmness was significantly positively correlated with sucrose and starch contents. Altogether, we uncovered the potential mechanism of chitosan coating mitigating melon softening and chilling injury through the regulation of starch and sucrose metabolism.

## 1 Introduction

Hami melon, with its unique flavor, sweet taste, and high nutritional value, is a popular horticultural crop in Xinjiang, Northwest China. The melon fruit is normally harvested in a high temperature and high humidity season, which promotes faster fruit ripening and softening, and in turn reduces the storage life of melon fruit (Bi et al., 2003; Liu et al., 2004). Softening, a complex process is usually associated with the degradation of fruit cell wall components involving an increase in water-soluble pectin (WSP) and a decrease in insoluble and covalently bound pectin (Zerpa-Catanho et al., 2017). Enzymes such as pectinesterase (PE; EC 3.1.11) and polygalacturonase (PG; EC 3.2.1.6.9) and related factors directly participate in fruit cell wall degradation (Deytieux-Belleau et al., 2008). Zhang et al. (2021) demonstrated that cold storage can delay the softening of melon fruit by regulating the transcript abundance of PE and PG genes. Cold storage is a widely used postharvest technology to maintain the quality and prolong the shelf life of fruit and vegetables. Although cold storage slows the decline in melon firmness, the fruit taste, especially sweetness, is negatively affected by long-term cold storage (Wang et al., 2020; Zhang et al., 2021). Furthermore, Hami melon, a cold-sensitive fruit, is susceptible to chilling injury when stored at low temperatures. Typical symptoms of chilling injury in Hami melons include discoloration, surface pitting, brown spots, and decay development (Bi et al., 2003; Fogelman et al., 2011). The development of chilling injury seriously reduces melon quality such as appearance, texture, flavor and nutrition (Zhang et al., 2017; Zhang et al., 2021). Therefore, novel practical methods alleviating chilling injury in melon are urgently needed.

Recent studies showed that edible coatings can preserve fruit quality and prolong the postharvest shelf life of cold-stored fruit by regulating the internal gas environment, reducing water loss, and delaying fruit ripening and senescence (Maringgal et al., 2020). Chitosan, a non-toxic high-molecular-weight cationic polysaccharide, is mainly obtained from the partial deacetylation of natural chitin (Romanazzi et al., 2017). Chitosan, with good biocompatibility, biodegradability, antibacterial activity, and film-forming ability, is widely used as a fruit preservation coating (Maringgal et al., 2020). Chitosan coating improves firmness and soluble solid content (SSC) in fruit (Maringgal et al., 2020; Chen et al., 2021). In guava, chitosan coating delayed fruit ripening and prolonged shelf life by enhancing antioxidant processes (Silva et al., 2018). Similarly, chitosan treatment was shown to retard the degradation of total soluble sugar and sucrose in post-harvested longans (Lin et al., 2020). However, the effects of chitosan treatment on fruit softening, chilling injury, and starch and sucrose metabolism in cold-stored melon are largely unclear.

Starch and sucrose metabolism not only affect fruit quality and sweetness but also are closely related to chilling tolerance during cold storage (Yu et al., 2017; Zhang et al., 2021). Sucrose, apart from being an energy source, also functions as an osmoregulator, cryoprotectant, reactive oxygen scavenger, and signaling molecule, contributing to cell membrane balance and antioxidant systems (Wang et al., 2019). The increase of sucrose level was correlated with the reduction of chilling injury and the enhancement of cold resistance (Zhao et al., 2022). The correlation between fruit quality and key enzymes of sucrose metabolism such as sucrose synthase (SS) and sucrose phosphate synthase (SPS) has been extensively studied (Chen et al., 2019; Duan et al., 2019). Researchers showed that exogenous ATP (Duan et al., 2019), sodium nitroprusside (Chen et al., 2019), and 6-benzylaminopurine (Luo et al., 2017) treatments can effectively maintain the fruit quality by regulating the activity of sucrose metabolism-related enzymes. Likewise, the exogenous application of salicylic acid (Zhao et al., 2021), abscisic acid (Zhao et al., 2022), and glycine betaine (Wang et al., 2019) can mitigate chilling injury by regulating sucrose metabolism in peach fruit.

Starch has multiple cellular functions, including in abiotic stress response (Thalmann and Santelia, 2017). In plants, altering starch structure and content are the two common ways to counter abiotic stress. Cold stress often triggers starch degradation (Dong and Beckles, 2019). β-amylase (BMY), a hydrolytic enzyme, converts starch into maltose and has been related to cold stress response (Zhao et al., 2019). A study showed that cold-tolerant banana cultivars have 3.0 times higher BMY activity than susceptible cultivars (Der Agopian et al., 2011). Cold stress response induced regulation of BMY genes expression promotes starch degradation and in turn increases soluble sugars in cold-stored melon (Zhang et al., 2021). Similarly, overexpression of PbrBMY3 promotes starch degradation and improves cold tolerance in pears (Zhao et al., 2019). Collectively, these studies suggest that regulating starch and sucrose metabolism is an important cold stress countering mechanism and must be investigated in detail. In addition, fruit softening has been linked to starch and sucrose metabolism in banana, mango, and kiwifruit (Nardozza et al., 2013; Zhu et al., 2021). During the process of blueberry fruit softening, the sucrose, glucose, and fructose contents changed accordingly (Wang et al., 2020). The blueberry firmness was positively correlated with sucrose content and SPS activity. However, a possible similar role of starch and sucrose metabolism in melon during postharvest cold storage is yet to be defined. Therefore, it is of great significance to explore the changes in starch and sucrose metabolism accompany the softening of melon, as well as the link between them.

At present, much remains to be determined concerning the regulation mechanism of chitosan treatment in melon fruit, including whether the effect of chitosan to mitigate chilling injury is related to starch and sucrose metabolism. Accordingly, we investigated the effects of chitosan treatment on fruit quality indices and starch and sucrose metabolism with the objective of providing a new perspective on the mechanism of chilling injury in melon fruit, and developing an eco-friendly, safe, and efficient postharvest technology to preserve the quality and prolong the shelf life of harvested melons.

## 2 Materials and methods

### 2.1 Melon fruit

“Gold Queen” (*Cucumis melo* L.) melons were picked from a farm in Shihezi, Xinjiang, China at commercial maturity (about 13% SSC). Fresh fruits of uniform shape, size, and color were selected and transported to the laboratory immediately after harvest.

### 2.2 Chitosan treatment and sample collection

Chitosan (deacetylated degree > 90%; viscosity 200 cP) was obtained from Rongna Biological Technology Co., Ltd. (Anhui, China). Chitosan solution (1.5%, w/v) was prepared as described in Silva et al. (2018). Briefly, 15 g of chitosan was dissolved in 1 L of distilled water with 15 mL of glacial acetic acid. Tween-80 and glycerol were added as emulsifiers.

The selected melons were randomly divided into two groups; the treatment group was immersed in 1.5% chitosan solution for 10 min, while the control group was treated with distilled water. The treated melons were air-dried and then stored at 3°C and 85-95% relative humidity (RH) for 30 d. Fruits were sampled following the method of Zhang et al. (2021) at 0, 6, 12, 18, 24, and 30 d of cold storage to measure fruit firmness and SSC. A part of the samples was snap-frozen in liquid nitrogen and then stored at −80°C for subsequent analysis.

### 2.3 Measurements of firmness, chilling injury, weight loss, and SSC

Fruit firmness (newtons, N) and chilling injury (%) were measured according to the method of Ning et al. (2019). To measure weight loss, fresh melon fruits were weighed at the beginning of the storage, and thereafter at every six-day interval (i.e., on the 6^th^, 12^th^, 18^th^, 24^th^, and 30^th^ d). Weight loss (%) was calculated as follows:


Weight loss=100×(initial weight-final weight)/initial weight

SSC (%) was measured using a digital saccharimeter (SW-32D, Guangzhou Suwei Electronic Technology Co., Ltd., China).

### 2.4 Extraction and determination of cell wall components

Cell-wall components including WSP, ionic-soluble pectin (ISP), and covalent-soluble pectin (CSP) were measured after extraction. Briefly, 1.0 g of melon was homogenized in 10 mL of 80% (v/v) ethanol and the mixture was boiled in a water bath at 95°C for 20 min. Afterward, the mixture was cooled and centrifuged at 4,000 x g at 25°C for 10 min. The obtained residue was washed twice in 15 mL of 80% (v/v) ethanol and acetone solution to clear starch and then dissolved in 10 mL of 90% (v/v) dimethyl sulfoxide for 15 h. Finally, according to the manufacturer’s instructions, WSP, ISP, and CSP were separated and determined using the micro method (WSP, ISP, and CSP content assay kit, Sino Best Biological Technology Co., Ltd, China).

### 2.5 Determination of fructose, glucose, sucrose, and starch content

Fructose, glucose, sucrose, and starch were extracted from the melon sample as described by Tao et al. (2021) and Luo et al. (2017) with minor modifications. Samples (1.0 g) are accurately weighed, homogenized in 10 mL distilled water, and then incubated in a water bath of 95°C for 10 min. After centrifugation of the mixture at 8,000 x g for 10 min at 25°C, the supernatant was collected for determination of glucose content.

Fructose and sucrose were extracted as follows. 1.0 g sample from the respective treatment was homogenized in 10 mL of 80% (v/v) ethanol, and then incubated in a water bath of 80°C for 10 min. After cooling, the homogenate was centrifuged at 4,000 x g for 10 min at 25°C. 2 mg activated carbon was added to the supernatant, and decolorization was performed at 80°C for 30 min. Then 1 mL of 80% (v/v) ethanol was added, and the mixture was centrifuged at 4,000 x g for 10 min at 25°C. The supernatant was collected.

Starch extraction: about 1.0 g of the sample was homogenized in 10 mL of 80% (v/v) ethanol, and then water bathed at 80°C for 30 min. After centrifugation at 3,000 x g for 5 min at 25°C, 5 mL of double-distilled water was added to the obtained residue, which was gelatinized in boiling water for 15 min. After cooling, 3.5 mL of 9.2 mol L^-1^ HClO_4_ was added to the residue, and extraction was performed at room temperature for 15 min. Then, 8.5 mL of double-distilled water was added and the mixture was centrifuged at 3,000 x g for 10 min at 25°C. The supernatant was diluted 4 times with distilled water for content determination.

Respective assay kits (Sino Best Biological Technology Co., Ltd, China) were used to determine the corresponding contents.

### 2.6 Enzymes activities

Melon samples (0.5 g) were ground in 4.5 mL 50 mM Tris-HCl buffer (pH 7.4) and the homogenate was centrifuged at 8000 x g for 15 min. The supernatant was collected for measuring enzyme activities. The activities (U mL^-1^) of PE, PG, SS, SPS, AMY, and BMY were determined using the corresponding enzyme-linked immunosorbent assay (ELISA) kits as reported by Zhang et al. (2021).

### 2.7 Gene expression analysis

Total RNA was extracted using the total RNA extraction kit having UNIQ-10 columns (a Trizol type system, Sangon Biotech, China) following the manufacturer’s instructions. The primers of selected genes were designed using the Primer Premier 5.0 software and shown in **Supplementary Table 1**. The quantitative real-time PCR (qRT-PCR) was performed using Fast SYBR Green Master Mix (BBI, China) on a LightCycler480 II System (Rotkreuz, Switzerland) as described previously (Zhang et al., 2021). The temperature program was as follows: 95°C for 3 min, 45 cycles of 95°C for 5 s, and then 60°C for 30 s. The relative expressions of genes were calculated using the 2^-ΔΔCt^ method (Livak and Schmittgen, 2001). Glyceraldehyde-3-phosphate dehydrogenase (GAPDH) was used as the reference gene.

### 2.8 Statistical analysis

All statistical differences and correlation analyses were performed using SPSS 17.0 (SPSS Inc., IL, USA) software. Data are presented as means ± standard errors; Data with *P*-value < 0.05 or < 0.01 were considered significant.

## 3 Results

### 3.1 Effects of chitosan treatment on the fruit firmness, weight loss, chilling injury, and SSC in postharvest cold-stored melon

**Figure 1:**
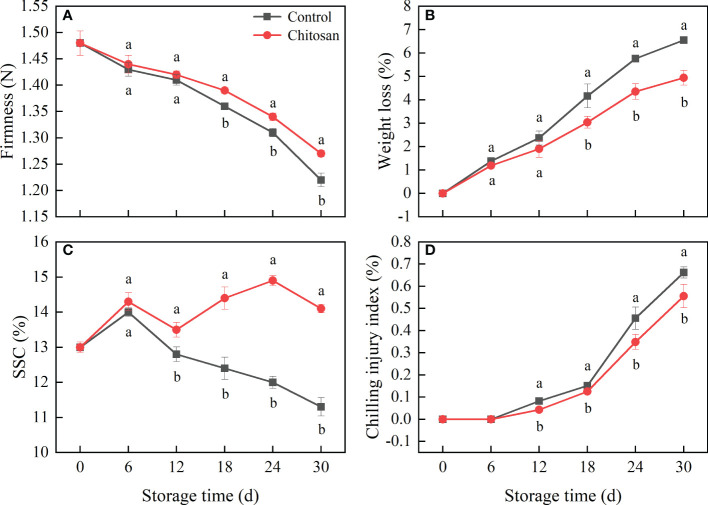
Effects of chitosan treatment on firmness **(A)**, weight loss **(B)**, SSC **(C)**, and chilling injury **(D)** in melon during storage at 3°C. Vertical bars represent the standard deviation (n = 3). Different letters on the same-day data point represent the significant differences between the control and chitosan-treated fruits (*P* < 0.05).

The fruit firmness in both the control and chitosan-treated melons decreased throughout the storage at 3°C (**Figure 1A**). Though chitosan treatment reduced the loss in fruit firmness, no notable difference was observed between the two groups until 12 d.

The weight loss of melon fruit increased continuously during the 30 d of storage (**Figure 1B**), however, chitosan coating treatment significantly reduced (*P* < 0.05) the weight loss after 18 d of storage, corresponding to 26.99%, 24.49%, and 24.52% decrease than that in control fruits on day 18, 24 and 30, respectively.

As shown in **Figure 1C**, SSC was higher in chitosan-treated melons than in control melons. In the control group, the highest SSC was 14.0% after 6 d of storage. In contrast, chitosan-treated fruits had a fluctuating trend of SSC, which first peaked on day 6 (14.3%), and then again on day 24 (14.9%).

The chilling injury appeared after 6 d of storage and then continued in both the control and chitosan-treated melons (**Figure 1D**). However, the damage from chilling injury was significantly lower in chitosan-treated fruits (*P* < 0.05) than in control fruits.

### 3.2 Effect of chitosan treatment on cell wall polysaccharide composition in postharvest cold-stored melon

**Figure 2:**
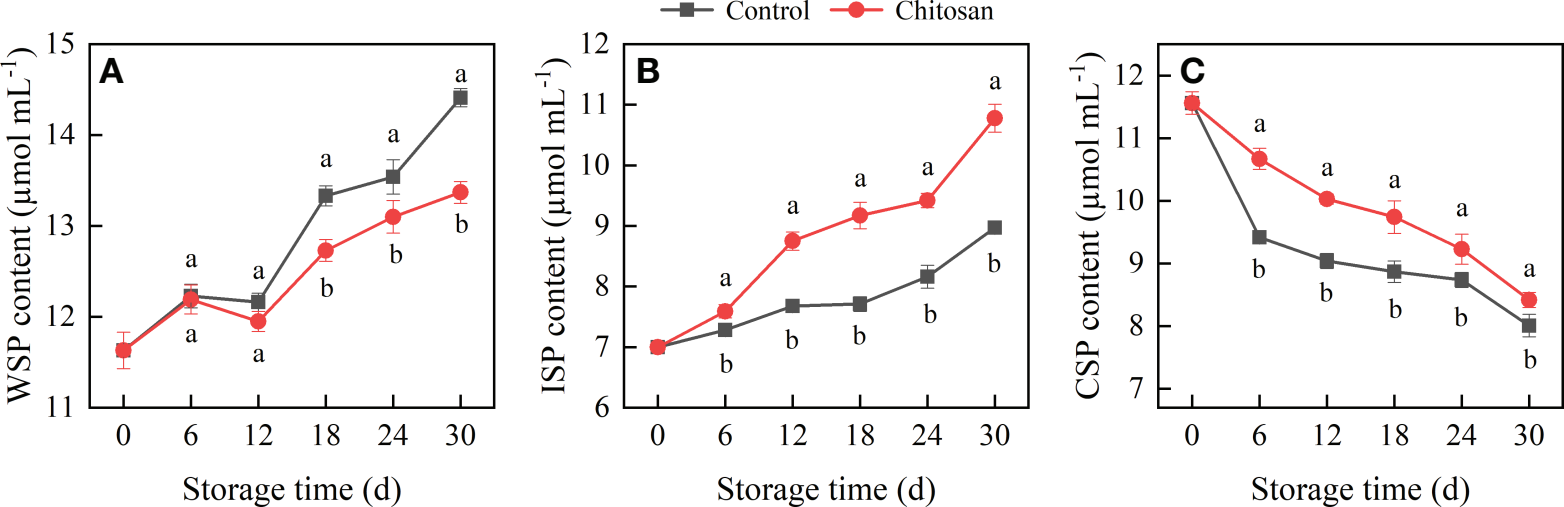
Effects of chitosan treatment on the WSP **(A)**, ISP **(B)**, and CSP **(C)** contents in postharvest cold-stored melon. Vertical bars represent the standard deviation (n = 3). Different letters on the same-day data point represent the significant differences between the control and chitosan-treated fruits (*P* < 0.05).

The WSP content in melons first increased, then decreased slowly at 6-12 d, and finally increased from 12 to 30 d in both groups (**Figure 2A**). However, compared with control fruits, chitosan-treated melons had lower levels of WSP throughout the storage period, especially significantly lower on 18, 24, and 30 d.

The ISP content in melons gradually increased during the entire storage period (**Figure 2B**). Surprisingly, chitosan treatment significantly increased the ISP content in melons from 6 to 30 d, which was 4.20% (day 6), 13.96% (day 12), 18.95% (day 18), 15.44% (day 24), and 20.16% (day 30) higher than that in control melons, respectively.

The CSP content in melons gradually decreased during the cold storage period (**Figure 2C**). In the control melons, the CSP content first decreased sharply between 0 to 6 days of storage and then declined slowly. Comparatively, chitosan-treated melons had a notably higher level of CSP throughout the storage period (*P* < 0.05).

### 3.3 Effects of chitosan treatment on enzyme activities and gene expressions related to melon fruit softening

**Figure 3:**
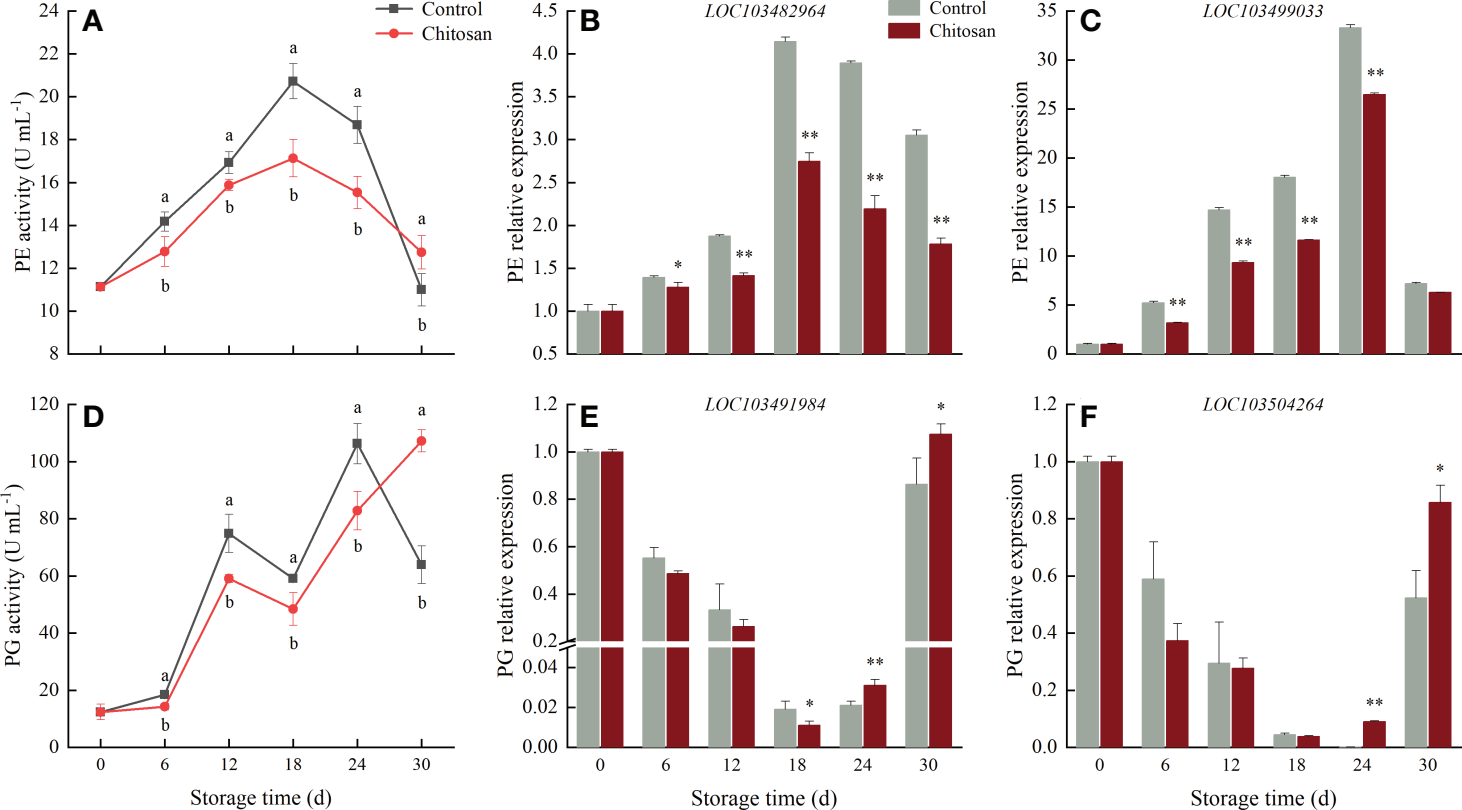
Effects of chitosan treatment on on the activities of PE **(A)**, PG **(D)** and gene expression levels of PE **(B)** *LOC103482964*, **(C)** *LOC103499033* and PG **(E)** *LOC103491984*, **(F)** *LOC103504264* in melon during storage at 3°C. Vertical bars represent the standard deviation (n = 3). Different letters on the same-day data point represent the significant differences between data (*P* < 0.05). * and ** indicate significant difference at *P* < 0.05 and *P* < 0.01.

During 30 d cold storage, the PE activity first increased to peak at 18 d, and then declined in both groups (**Figure 3A**). However, the PE activity in chitosan-treated melons always remained lower than in control melons (6 to 24 d). In the last 6 days of storage, the PE activity in control melons decreased rapidly and was significantly (*P* < 0.05) lower than in chitosan-treated melons at 30 d.

The PG activity in melons showed a fluctuating upward trend during the 30 d cold storage (**Figure 3D**). Post-harvest, the PG activity slightly increased until day 6, showing a significant (*P* < 0.05) difference between chitosan-treated and control fruits. Afterward, the trend changed rapidly, and the PG activity in chitosan-treated melons turned significantly (*P* < 0.05) lower than in control melons, except on the day 30^th^.

As shown in **Figures 3B, C**, the expression of two PE genes (*LOC103482964* and *LOC103499033*) increased until day 18 in control fruits and day 24 in chitosan-treated fruits and then decreased. Notably, throughout the storage period, the expression of PE genes was lower in chitosan-treated fruits than in control fruits. During the 30 d cold storage, the expression of two PG genes, *LOC103491984* and *LOC103504264*, gradually decreased to reach the minimum on the 18^th^ and 24^th^ d, respectively, and then increased in both groups (**Figures 3E, F**). In addition, both PG genes were downregulated in chitosan-treated fruits during 6-24 d compared with control fruits.

### 3.4 Effect of chitosan treatment on the starch, sucrose, glucose, and fructose contents in postharvest cold-stored melon

**Figure 4:**
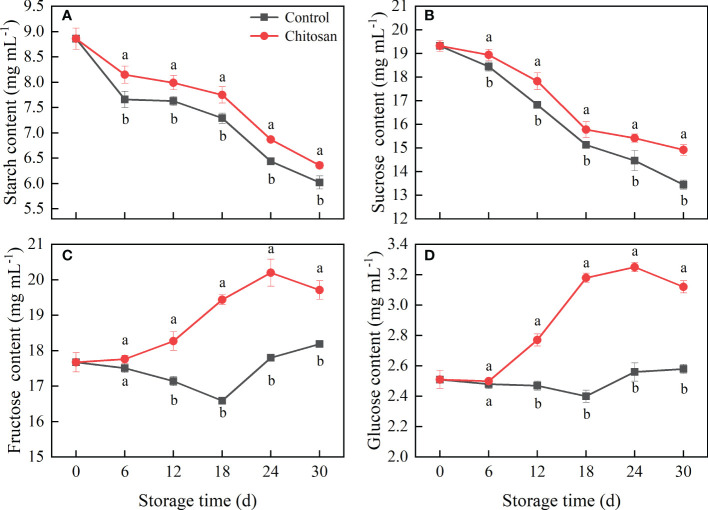
Effects of chitosan treatment on the starch **(A)**, sucrose **(B)**, fructose **(C)**, and glucose **(D)** contents in postharvest cold-stored melon. Vertical bars represent the standard deviation (n = 3). Different letters on the same-day data point represent the significant differences between data (*P* < 0.05).

The influence of chitosan treatment on the contents of starch, sucrose, glucose, and fructose in cold-stored melons is shown in **Figure 4**. The trends of change in starch and sucrose contents were alike, which began to decline from day zero (**Figures 4A, B**). However, post-harvest chitosan treatment significantly inhibited the degradation of starch and sucrose during the entire storage period (*P* < 0.05). The trends of change in fructose and glucose contents were also similar in both groups (**Figures 4C, D**), however, the fructose and glucose contents were higher in the chitosan-treated melons. The highest fructose and glucose levels were on day 24^th^, which were 13.45% and 26.81% higher in chitosan-treated fruits than those in control, respectively.

### 3.5 Effect of chitosan treatment on enzyme activities and gene expressions related to starch and sucrose metabolism

**Figure 5:**
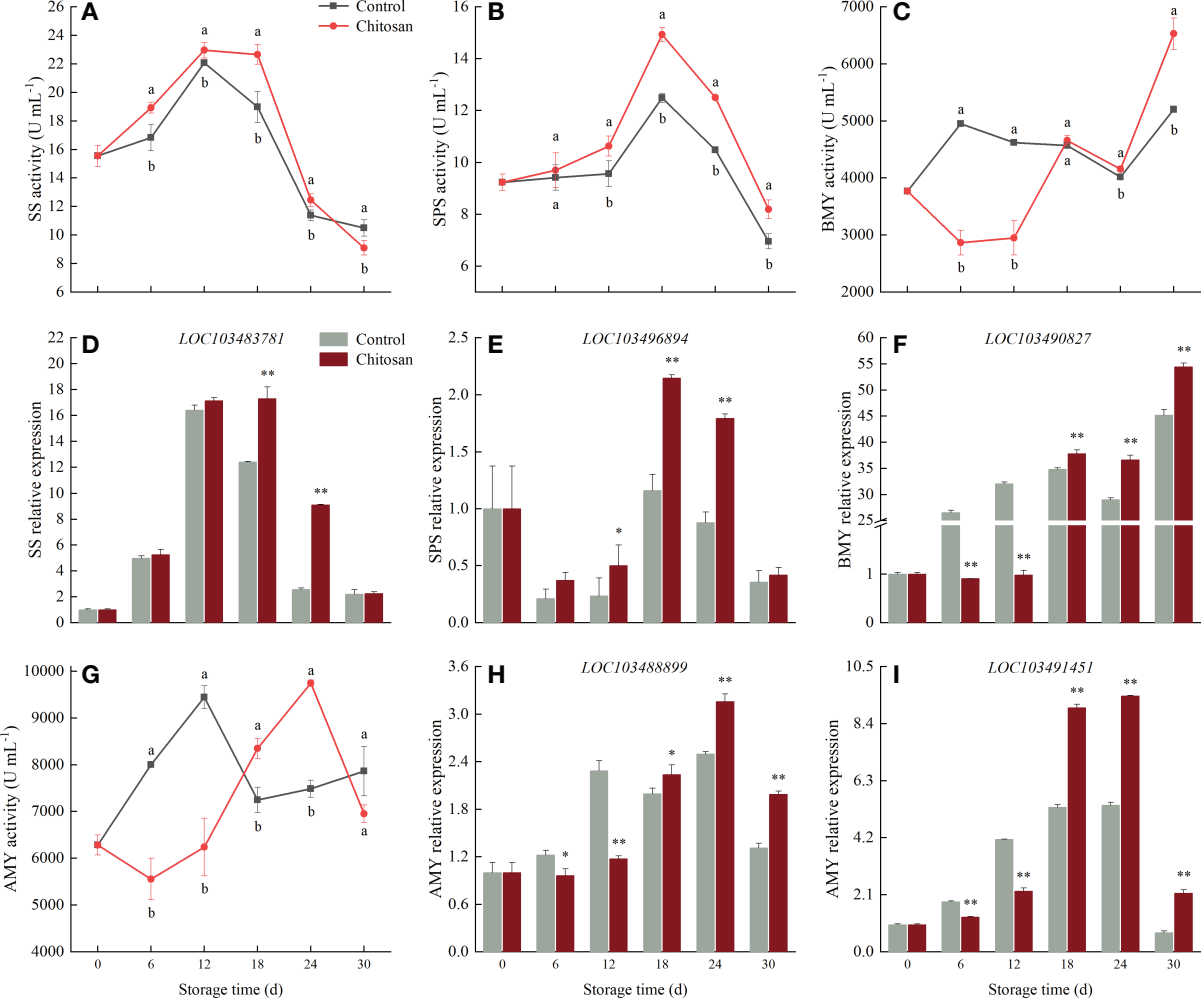
Effects of chitosan treatment on the activities of SS **(A)**, SPS **(B)**, BMY **(C)**, AMY **(G)**, and gene expression levels of SS **(D)** *LOC103483781*, SPS **(E)** *LOC103496894*, BMY **(F)** *LOC103490827*, and AMY **(H)** *LOC103488899*, **(I)** *LOC103491451* in melon during storage at 3°C. Vertical bars show the standard deviation (n = 3). Different letters on the same-day data point indicate significant differences between data (*P* < 0.05). * and ** indicate significant difference at *P* < 0.05 and *P* < 0.01.

The changes in the enzyme activities and corresponding genes of SS, SPS, AMY, and BMY in cold-stored melons are shown in **Figure 5**. SS and SPS activities first increased and then declined, reaching the maximum on the 12^th^ and 18^th^ d, respectively (**Figures 5A, B**). The SS activity did not vary greatly in chitosan-treated fruits but decreased rapidly in control fruits during 12-18 d of storage (**Figure 5A**); the SS activity was 19.42% higher in chitosan-treated melons than that in control on the 18^th^ day. As shown in **Figure 5B**, chitosan treatment promoted SPS activity, which increased until the 18^th^ d, and was significantly (*P* < 0.05) higher than in control fruits, except on the 6^th^ d. Correspondingly, the *SS* (*LOC103483781*) and *SPS* (*LOC103496894*) genes were upregulated in chitosan-treated melons during the 6-30 d of cold storage (**Figures 5D, E**).

The activity and expression level of BMY in control fruits were higher than in chitosan-treated fruits between 6 to 12 d of cold storage (**Figures 5C, F**). However, after the 18^th^ d onwards, chitosan treatment enhanced BMY activity and gene expression and maintained them at a high level.

AMY activity fluctuated in control and chitosan-treated fruits (**Figure 5G**). In the early stage of storage (6-12 d), control fruits displayed a higher level of AMY activity than chitosan-treated fruits. However, in the middle and late stages of cold storage (18-24 d), the AMY activity significantly increased in chitosan-treated fruits (*P* < 0.05) along with AMY gene expression (**Figures 5H, I**).

### 3.6 Correlation analysis

The correlations between fruit firmness, cell wall degradation parameters, and starch and sucrose metabolism indices were analyzed in chitosan-treated and control fruits (**Table 1**). Fruit firmness was significantly positively correlated with starch content, sucrose content, and SS activity (*P* < 0.01). Starch and sucrose contents were positively correlated with the CSP content and negatively correlated with PG activity and WSP and ISP contents (*P* < 0.01) in both fruits. Softening indices showed a highly negative correlation with the glucose and fructose contents in chitosan-treated melons (*P* < 0.05). SS activity was positively correlated with CSP content and PE activity (0.20 < r^2^ < 0.52), and negatively correlated with WSP and ISP contents and PG activity (-0.62 < r^2^ < -0.22) in both types of fruits. SPS activity was significantly positively correlated with PE activity (*P* < 0.01). AMY and BMY activities were positively correlated with WSP and ISP contents in both groups; however, the correlation was more significant in chitosan-treated fruits. A highly significant positive correlation of AMY activity was observed with PE and PG activities (*P* < 0.05) and between BMY and PG activities in chitosan-treated fruits (*P* < 0.01). Altogether, correlation analysis revealed that melon fruit softening is associated with starch and sucrose metabolism, which can affect the quality of cold-stored melons.

**Table 1:** Correlation analysis of firmness with call wall degradation parameters and starch and sucrose metabolism indices in chitosan-treated and control melons.

Chitosan-treated melon fruit						
Index	Firmness	WSP content	ISP content	CSP content	PE activity	PG activity
Starch content	0.971^**^	-0.936^**^	-0.932^**^	0.948^**^	-0.261	-0.925^**^
Sucrose content	0.902^**^	-0.924^**^	-0.917^**^	0.910^**^	-0.482^*^	-0.880^**^
Glucose content	-0.799^**^	0.838^**^	0.840^**^	-0.836^**^	0.613^**^	0.811^**^
Fructose content	-0.812^**^	0.869^**^	0.805^**^	-0.836^**^	0.475^*^	0.813^**^
SS activity	0.610^**^	-0.562^*^	-0.428	0.447	0.509^*^	-0.537^*^
SPS activity	0.027	0.167	0.099	-0.114	0.821^**^	-0.011
AMY activity	-0.483^*^	0.589^*^	0.474^*^	-0.492^*^	0.552^*^	0.498^*^
BMY activity	-0.810^**^	0.751^**^	0.780^**^	-0.708^**^	-0.056	0.721^**^
Control melon fruit						
Starch content	0.945**	-0.924^**^	-0.914^**^	0.919^**^	-0.212	-0.721^**^
Sucrose content	0.940^**^	-0.944^**^	-0.919^**^	0.856^**^	-0.304	-0.771^**^
Glucose content	-0.441	0.302	0.496^*^	-0.152	-0.468^*^	0.261
Fructose content	-0.389	0.279	0.446	-0.060	-0.706^**^	0.034
SS activity	0.624^**^	-0.614^**^	-0.580^*^	0.225	0.383	-0.202
SPS activity	0.316	-0.160	-0.413	0.070	0.859^**^	0.171
AMY activity	-0.135	0.024	0.227	-0.513^*^	0.219	0.330
BMY activity	-0.495^*^	0.462	0.513^*^	-0.686^**^	-0.138	-0.011

* and ** indicate significant difference at P < 0.05 and P < 0.01.

## 4 Discussion

The edible coating is a modified atmosphere method that has been applied to preserve the quality of various fruit, such as tropical fruit, citrus, melon, tomato, and pome fruit (Maringgal et al., 2020). Chitosan, a promising coating material, has been widely used for food preservation and packaging; it is non-toxic, biodegradable, biocompatible, film-forming, and easily available (Maringgal et al., 2020; Kumarihami et al., 2022). Chitosan coating can reduce weight loss, respiration, and softening and increase SSC in post-harvest fruits (Huang et al., 2017; Kumarihami et al., 2020). In our study too, chitosan treatment produced similar results, reducing weight loss, softening, and chilling injury in cold-stored melons. Additionally, the degradation of starch and sucrose was also reduced. We found that chitosan treatment regulated the activities of starch and sucrose metabolism-related enzymes and the expression of corresponding genes.

Fruit softening is a crucial parameter that determines the length of the storage period and commercial value of post-harvest fruit. In our study, chitosan treatment effectively delayed the firmness loss in melons during 30 d of cold storage (**Figure 1A**). Meanwhile, WSP and ISP contents increased and CSP content decreased accompanying the softening of melon (**Figure 2**). Pectin, a structurally complex polysaccharide, is commonly related to fruit cell wall degradation and softening (Brummell et al., 2004). During fruit softening, insoluble pectin is converted to soluble pectin (Wang et al., 2020). The same was observed in our study. Pectin solubilization is associated with PE and PG enzyme activities (Wang et al., 2020; Win et al., 2021). The main function of PE is to catalyze the demethylation of pectin to generate low methyl-esterified pectin, which makes the cell wall easily decomposed by PG (Khademi et al., 2014). PG further hydrolyzes the galacturonide bond in the main chain of polygalacturonic acid, increasing pectin degradation and in turn fruit softening (Brummell et al., 2004). The correlation between fruit softening and cell wall metabolism-related enzymes has been investigated in many fruits (Lin et al., 2019; Chen et al., 2021). In our study, compared with PE activity, PG activity showed a stronger correlation with softening-related parameters, such as fruit firmness and WSP, ISP, and CSP contents (**Supplementary Table 2**). This suggests that PG plays a more important role than PE in fruit softening in melon under cold conditions.

Chitosan treatment was shown to delay fruit softening in many fruits (Kumarihami et al., 2022). Chitosan coating forms a protective cover on the fruit surface, which reduces gas exchange and moisture loss, thereby retarding the degradation of cell wall components and retaining fruit firmness (Maringgal et al., 2020; Kumarihami et al., 2022). In our study, chitosan treatment significantly slowed down the degradation of pectin in melon. Consistent with the previous findings (Lin et al., 2019), chitosan-treated fruits had a higher CSP content and lower WSP content than control, which improved fruit quality (Zhao et al., 2018; Lin et al., 2019). ISP, a form of pectin, is involved in fruit ripening. In longan fruit, the ISP content decreased, and the chitosan treated-longan showed higher ISP levels (Lin et al., 2019). However, the opposite change in ISP content was observed in our study (**Figure 2B**). The causes of these differences may be attributed to different roles of ISP in different stages of fruit ripening and senescence in different fruits, and demands further examination. A lower PE and PG activity was observed in chitosan-treated melons compared with control fruits during 0-24 d of cold storage. Although the PE and PG genes showed different expression patterns during storage, their expression was significantly inhibited by chitosan coating (**Figure 3**). Notably, the expression levels of PE and PG genes are closely related to the firmness of Hami melon (Zhang et al., 2021). Our results indicated that chitosan treatment reduced the enzyme activities and gene expression levels of PE and PG, which reduced the degradation of CSP and maintained the storability of cold-stored melons. This also suggests that pectin degradation contributes to the loss of fruit firmness in melon, which can be countered/regulated by chitosan coating.

Accompany the melon softening, the sucrose and starch content gradually changes. Starch and sucrose metabolism in fruit is the main factor regulating soluble sugar content, which causes the change in SSC, an important sensory parameter of fruit quality. Chitosan treatment could be used to preserve higher SSC in some fruits such as longan (Lin et al., 2020) and orange (Gao et al., 2018). Our result showed that chitosan treatment also increased the SSC compared with the control melon during the entire storage (**Figure 1C**). Potentially, chitosan coating treatment reduced respiration and transpiration rate, consequently retarding the loss of SSC and fruit ripening.

Sucrose content influences the quality, taste, and membrane stability of fruits (Wang et al., 2019; Zhao et al., 2021). Therefore, the regulation of sucrose metabolism through related enzymes is important in maintaining the quality of post-harvest fruits (Stadler et al., 1999). SS and SPS are the two key rate-limiting enzymes that transform fructose and glucose into sucrose (Wang et al., 2020; Zhang et al., 2021). We found that chitosan treatment increased the activities of SS and SPS in cold-stored melons (**Figures 5A, B**), which is consistent with the findings in peach fruit treated with glycine betaine (Wang et al., 2019) and 1-methycyclopropene (Yu et al., 2017). These results suggest that sucrose accumulation in melon was due to the increased activities of SS and SPS. In addition to sucrose accumulation, numerous studies have shown that the improved SS and SPS activities can also lead to the decomposition of fructose and glucose (Sun et al., 2020). Interestingly, in our study, the fructose and glucose contents in chitosan-treated melons were higher than those in control melons, which can be related to the decreased respiration of melons and the degradation of other carbohydrates such as starch.

Starch and sucrose are also key signaling molecules in plant stress response. The relationship between chilling injury and sugar content has been confirmed in several fruits (Wang et al., 2019; Zhao et al., 2021; Zhao et al., 2022). The degradation of starch or accumulation of sucrose has been related to enhanced tolerance to chilling injury under cold stress (Thalmann and Santelia, 2017; Zhao et al., 2021). Der Agopian et al. (2011) found that regulation of starch-to-sucrose metabolism is vital for cold acclimation in banana; increased starch mobilization to sucrose improved cold resistance. Starch is the major storage metabolite in plants, and its degradation is often induced by cold stress (Dong and Beckles, 2019). SS, SPS, AMY, and BMY are the major enzymes involved in the starch and sucrose metabolism in Hami melon (Zhang et al., 2021). AMY, an endo-amylolytic enzyme, cleaves the α-1,4 glycosidic bonds of starch, accelerating starch-to-sucrose metabolism (Der Agopian et al., 2011). SPS also contributes starch to sucrose conversion. BMY, a mediator of starch degradation to downstream sugars, is activated under cold stress (Zhao et al., 2019). We found that the activities of SPS, AMY, and BMY first increased and then decreased in cold-stored melons (**Figures 5A, C, G**), indicating that cold conditions had a significant regulatory effect on starch-to-sucrose metabolism in melon.

Chitosan treatment has been reported to reduce starch degradation in harvested fruits. Cosme Silva et al. (2017) observed a higher starch content in chitosan-treated mango. In our study, chitosan coating inhibited starch degradation and had different effects on the activities of AMY and BMY at different stages of 30 d cold storage in melon (**Figures 5C, G**). Possibly, chitosan coating slowed down transpiration and suppressed the respiration rate in melon. The inhibition of respiration rate coupled with reduced metabolic activity reduces the synthesis and utilization of metabolites, thereby delaying the degradation of starch to sugars (Kumarihami et al., 2022). Similar results were found in kiwifruit (Kumarihami et al., 2021). Our study showed that chitosan coating could alleviate chilling injury by enhancing AMY and BMY activities and accelerating starch degradation in melon during the later stages of cold storage.

Transcriptional regulations play an important role in regulating starch and sucrose metabolism under cold stress. Cold stress increases the expression of several genes related to sucrose and starch metabolism, which improves cold stress resistance (Thalmann and Santelia, 2017; Zhang et al., 2021). In this study, SS (*LOC103483781*) and SPS (*LOC103496894*) genes were upregulated by chitosan treatment, resulting in a higher content of sucrose in treated fruit (**Figures 5D, E**), which improves membrane stabilization and cold stress signaling (Zhao et al., 2021; Zhao et al., 2022). Under cold stress, soluble sugars can directly modulate gene expression by mediating sugar signaling pathways (Couee et al., 2006). Thus, chitosan coating-mediated reduction in loss of sucrose content can be the possible mechanism of countering chilling injury in cold-stored melons. Also, BMY (*LOC103490827*) and AMY (*LOC103488899* and *LOC103491451*) genes were upregulated in chitosan-treated melon during the later stage of cold storage (**Figures 5F, H, I**). Silencing of the *BMY* gene was shown to increase starch accumulation and decrease soluble sugar levels (Zhao et al., 2019). For example, abscisic acid treatment induced the expression of *BMY1* and *AMY3* genes in Arabidopsis, increasing starch degradation (Thalmann et al., 2016). Similar results were observed in our study (**Figure 5C**), suggesting that chitosan treatment mitigates chilling injury in melon fruit, at least in part, by upregulating *AMY* and *BMY* genes.

In addition to chilling injury, sucrose and starch metabolism are often related to fruit softening (Wang et al., 2020; Zhang et al., 2021). We found a significant positive correlation between melon fruit firmness and sucrose and starch contents (**Table 1**). Similar results were seen in blueberry fruit treated with ethylene (Wang et al., 2020). In our study, the correlations between starch content and firmness, WSP, ISP and CSP content in chitosan-treated fruit were higher than those in control group. However, opposite results were found in the correlation between sucrose content and fruit softening indicators. Thus, we speculate that the mechanism of chitosan delaying loss in melon firmness maybe firstly regulate the changes of starch metabolism. PE activity was positively correlated with SS activity, while PG and SS activities were negatively correlated. Moreover, in chitosan-treated cold-stored melons, a stronger correlation was observed between PG and the contents of starch, sucrose, glucose, and fructose than PE (**Table 1**). This could be another point to confirm the aforementioned hypothesis that PG plays a more important role in the softening of cold-stored melon fruit.

The correlation results revealed a certain link between fruit softening and sucrose and starch metabolism in melon during cold storage. These two complex processes involve multiple regulators including metabolites, enzyme activities, and gene regulations, which need to be investigated in future studies.

## 5 Conclusion

Our results showed that chitosan coating effectively reduced the conversion of CSP, inhibited the increase of WSP content, and maintained fruit firmness during cold storage of melon at 3°C. Moreover, PE and PG activities were lowered along with the downregulation of related genes. Our results also concluded that chitosan treatment alleviated chilling injury in cold-stored melons by regulating starch and sucrose metabolism. In addition, a strong correlation between melon fruit softening and starch and sucrose metabolism was observed. Altogether, chitosan coating could be a reliable and potential preservation technology for mitigating chilling injury and extending the postharvest shelf life of cold-stored melons. However, further studies are needed to comprehensively elucidate the other possible mechanisms of enhance chilling tolerance after chitosan treatment.

## Data availability statement

The original contributions presented in the study are included in the article/**Supplementary material**. Further inquiries can be directed to the corresponding author.

## Author contributions

QZ: Data curation, software, formal analysis, writing - original draft, writing - review & editing. FT: Methodology, project administration. WC: Software. BP: Validation. MN: Validation. CS: Funding acquisition, supervision, visualization. XY: Investigation, conceptualization. All authors contributed to the article and approved the submitted version.
