# Supplementary material for: Chitosan treatment reduces softening and chilling injury in cold-stored Hami melon by regulating starch and sucrose metabolism
Source: Front Plant Sci. 2022 Dec 14;13:1096017. doi: 10.3389/fpls.2022.1096017 (PMC9795072; doi:10.3389/fpls.2022.1096017)
Supplement: Supplementary file 1 [file Table_1.doc]

Supplementary Table 1The sequences of speciﬁc primers used for qRT-PCR analysis

| Gene ID | Forward primer (5′to 3′) | Reverse primer (5′to 3′) | Product (bp) |
| --- | --- | --- | --- |
| LOC103482964 | TCCGTCGTCGCTCTCGTATCC | GGCTTGGGAGGGCTTGTTGTG | 143 |
| LOC103499033 | TCGCCACCTGCTCCTCCATC | GCGGCAACAACCACCATACCC | 131 |
| LOC103491984 | GACTGCTGCTGCTGGTGGATTG | ACGGTAGACGCAACTGTAGAGGAG | 132 |
| LOC103483871 | CCCAGATTCCTCCGATCATGTATGC | TCCCAACCACTCTTCAAGGCAATG | 84 |
| LOC103488899 | GCTACTGCTATCTCTGCTTGTGCTG | AACCGTCCTGCTGTATGAATGCC | 82 |
| LOC103491451 | GGGTTTCCTTGGTCCTGGCAAAG | ACATTCCGAGCAGAACGAAGAGAAC | 132 |
| LOC103490827 | TGAACAAACCTCGGGCGATGAATG | CCACCAAGCATCCACCATAACTCC | 83 |
| LOC103483781 | AACCGCCACAACTTCACCACAG | TGCCCAACCTCCACCGAACC | 117 |
| LOC103496894 | TGCGACCGCCATTGAAGAACAG | GTCCGATTCATCCACACCACTAACC | 127 |

Supplementary Table 2 The analysis of correlation between call wall degradation indicators of chitosan-treated and control melon fruit.

|  | **Chitosan-treated melon fruit** | | | | | | |
| --- | --- | --- | --- | --- | --- | --- | --- |
|  | Index | Firmness | WSP content | ISP content | CSP content | PE activity | PG activity |
|  | Firmness | 1 | -0.913^**^ | -0.945^**^ | 0.948^**^ | -0.201 | -0.936^**^ |
|  | WSP content | -0.913^**^ | 1 | 0.874^**^ | -0.894^**^ | 0.286 | 0.816^**^ |
|  | ISP content | -0.945^**^ | 0.874^**^ | 1 | -0.978^**^ | 0.391 | 0.954^**^ |
|  | CSP content | 0.948^**^ | -0.894^**^ | -0.978^**^ | 1 | -0.395 | -0.942^**^ |
|  | PE activity | -0.201 | 0.286 | 0.391 | -0.395 | 1 | 0.293 |
|  | PG activity | -0.936^**^ | 0.816^**^ | 0.954^**^ | -.942^**^ | 0.293 | 1 |
|  |  |  |  |  |  |  |  |
|  | **Control melon fruit** | | | | | | |
|  | Firmness | 1 | -0.963^**^ | -0.969^**^ | 0.824^**^ | -0.027 | -0.628^**^ |
|  | WSP content | -0.963^**^ | 1 | 0.917^**^ | -0.817^**^ | 0.133 | 0.589^*^ |
|  | ISP content | -0.969^**^ | 0.917^**^ | 1 | -0.819^**^ | -0.042 | 0.632^**^ |
|  | CSP content | 0.824^**^ | -0.817^**^ | -0.819^**^ | 1 | -0.356 | -0.681^**^ |
|  | PE activity | -0.027 | 0.133 | -0.042 | -0.356 | 1 | 0.568^*^ |
|  | PG activity | -0.628^**^ | 0.589^*^ | 0.632^**^ | -0.681^**^ | 0.568^*^ | 1 |
